# Overexpressed Smurf1 is degraded in glioblastoma cells through autophagy in a p62‐dependent manner

**DOI:** 10.1002/2211-5463.13310

**Published:** 2021-11-15

**Authors:** Da Han, Shengzhen Li, Qin Xia, Xinyi Meng, Lei Dong

**Affiliations:** ^1^ School of Life Science Beijing Institute of Technology China

**Keywords:** autophagy, degradation, E3 ubiquitin ligase activity, p62, PI3K/Akt signaling, Smurf1

## Abstract

Homologous to E6AP C‐terminus (HECT)‐type E3 ubiquitin ligase SMAD‐specific E3 ubiquitin protein ligase 1 (Smurf1) was originally identified to ubiquitinate Smad protein in the TGF‐β/BMP signaling pathway. Recently, Smurf1 has been reported to promote tumorigenesis by regulating multiple biological processes. High expression of Smurf1 plays a vital role in brain tumor progression by mediating aberrant cell signaling pathways. Previous reports have shown that Smurf1 is degraded mainly through the ubiquitin–proteasome system, but it remains unclear whether Smurf1 is degraded by autophagy in tumor cells. In this study, we show that autophagy activators promote Smurf1 degradation in glioblastoma (GB) cells. The autophagy receptor p62 colocalizes with ubiquitinated substrates to promote sequestration of cytoplasm cargo into the autophagosome. We report that autophagic degradation of Smurf1 is dependent on p62. Moreover, the autophagic degradation of Smurf1 is prevented in the absence of the HECT domain or E3 ubiquitin ligase activity. We further proved that activation of autophagy leads to a decrease of Smurf1 and the inhibition of the phosphoinositide 3‐kinase/protein kinase B signaling pathway in GB cells. Our results suggest that enhancement of autophagic degradation of Smurf1 may be a potential approach to treating GB.

## Graphical abstract

In this article, we report that oncogenic SMAD‐specific E3 ubiquitin protein ligase 1 is degraded by autophagy in a p62 and E3 ubiquitin ligase activity‐dependent manner in glioblastoma cells, a finding that was not previously described. Our research identifies a novel degradation pathway for tumor‐related proteins, which has certain guiding significance for tumor treatment.

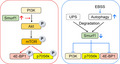

**Abbreviations**
CS: codon‐switched
GB: glioblastoma
Nrf2: nuclear factor erythroid 2‐related factor 2
NS: not significant
PI3K/Akt: phosphoinositide 3‐kinase/protein kinase B
SD: standard deviation
Smurf1: SMAD‐specific E3 ubiquitin protein ligase 1
UPS: ubiquitin–proteasome system

The aberrantly expressed SMAD‐specific E3 ubiquitin protein ligase 1 (Smurf1) has been reported to play a pivotal role in multiple tumor types [1, 2, 3]. Overexpressed Smurf1 promotes tumorigenesis through targeting tumor suppressor RhoB for ubiquitination degradation in cervical carcinoma [4]. In ovarian cancer, aberrantly activated Smurf1‐mediated degradation of tumor suppressor ARHGAP26 promotes invasion and metastasis of cancer cells through the β‐catenin pathway [5]. Smurf1 supports breast cancer growth through facilitating estrogen receptor α signaling, which is necessary for estrogen‐dependent cancer progression [6]. The suppression of Smurf1 inhibits the progression of multiple types of cancer. Down‐regulation of Smurf1 through microRNA shows gastric cancer‐suppressing effect [7]. Moreover, overexpressed Smurf1 plays a pivotal role in brain tumor. For example, hyperactivation of Smurf1 is positively correlated with the World Health Organization grade of gliomas [8]. A recent study demonstrates that Smurf1 knockdown reduces glioblastoma (GB) progression through ubiquitination of PTEN [9]. The oncogenic role of Smurf1 is associated with the activation of phosphoinositide 3‐kinase/protein kinase B (PI3K/Akt) signaling [10]. However, the degradation and the aberrant expression of Smurf1 remain largely unknown.

The ubiquitin–proteasome system (UPS) and autophagy are two major quality‐control systems for self‐regulation and degradation of proteins/organelles in eukaryotic cells [11]. UPS degrades ubiquitin‐tagged redundant proteins by 26S proteasome [12]. Previous studies have demonstrated that Smurf1 is degraded via UPS [13]; however, it has not been elucidated whether autophagy regulates Smurf1 degradation. Autophagy is a highly conserved eukaryotic cellular recycling process that is pivotal for maintaining cellular homeostasis, which delivers damage or excess organelles/proteins to lysosomes for recycling in eukaryotic cells [14]. Among the multiple subtypes, macroautophagy (thereafter referred to as autophagy) is well characterized. It requires the formation of autophagosome, which is a double‐membrane vacuole that packages the sequestered redundant cytoplasmic cargoes, and the fusion of autophagosome and lysosome for degradation of cellular cargo [15]. Autophagy is not only a nonselective bulk degradation pathway for cytoplasmic cargo but also a selective pathway involved in several autophagy receptors [16]. In the process of autophagy, p62/SQSTM1 (sequestosome 1) plays an important role as a selective autophagy receptor to target ubiquitinated cytoplasm cargo for autophagic degradation [17, 18]. It binds to LC3 on the phagophore and ubiquitinated cytoplasm cargo, thereby mediating the selective autophagy degradation of the specific substrates [19]. Recently, E3 ubiquitin ligase has been reported to interact with p62. The interaction between E3 ligase NEDD4 and p62 is reported to promote p62‐mediated autophagy [20, 21]. Kelch‐like ECH‐associated protein 1 (KEAP1), which is a component of the Cullin 3 (CUL3)‐based E3 ubiquitin ligase complex, binds to phosphorylated p62 for autophagic degradation [22]. Whether E3 ubiquitin ligase Smurf1 interacts with p62 and is degraded by autophagy in tumor cells remains elusive.

In this study, we hypothesized that Smurf1 is degraded through autophagy in a p62‐dependent manner, and the autophagic degradation of Smurf1 inhibits PI3K/Akt signaling in GB cells. To better understand the degradation pathway of Smurf1, we treated GB cell lines by autophagy activators with or without UPS inhibitor. We also performed RNA interference and immunofluorescence to check the role of p62 in Smurf1 degradation. E3 ligase activity‐null mutant of Smurf1 and deletion constructs were used to check whether E3 ubiquitin ligase activity is required. We further performed autophagy activator treatment combined with overexpression of Smurf1 to detect the effect of autophagy degradation of Smurf1 on the PI3K/Akt signaling pathway. Because PI3K/Akt signaling is related to tumor cell progression, whether autophagy degrades overexpressed Smurf1 to inhibit the tumor growth is worth further investigation.

## Materials and methods

### Ethics statement

GB patient tissues were collected from Sanbo Brain Hospital, Capital Medical University. Written informed consent was obtained from each subject. All experiments conformed to the Declaration of Helsinki, and the study was performed after agreement from the Experimental Animal Protection and Ethics Committee, Beijing Institute of Technology.

### Reagents

Cells were seeded in 12‐well plates at approximately 80% confluence and grown for 24 h before treatment with proteasome inhibitor MG132 (20 mm, 12 h; HY‐13259; MCE, Monmouth Junction, NJ, USA), autophagy activator Torin1 (250 nm, 2 h; 4247; Tocris, Minneapolis, MN, USA), autophagy activator rapamycin (100 μm, 24 h; S1039; Selleck, Houston, TX, USA), Earle''s Balanced Salt Solution (EBSS) (2 or 3 h; 14155063; Gibco, Carlsbad, CA, USA) or autophagy inhibitor Bafilomycin A1 (100 nm, 24 h; 196000; Sigma, St. Louis, MO, USA).

### Cell culture and transfections

HEK293, U251 and LN229 cell lines were purchased from American Type Culture Collection (Baltimore, MD, USA). Primary GB cells were from Sun Yat‐sen University. All cells were cultured in Dulbecco's modified Eagle’s medium (C11995500BT; Gibco) supplemented with 10% FBS (F7524; Sigma) and 1% penicillin/streptomycin (100 U·mL^−1^ penicillin; 10 μg·mL^−1^ streptomycin) at 37 °C in a humidified 5% CO_2_ incubator.

The reagent for the plasmid transfection experiment was Lipofectamine 2000 (Invitrogen, Carlsbad, CA, USA). Twelve‐well plates were used for transfection. The transfection process followed the user's manual; 1.5 μg plasmids was transfected to each well.

### Quantitative real‐time PCR

TRNzol Universal (DP424; Tiangen, Beijing, China) was used to extract total RNA from LN229 cells treated with or without autophagy activators. The RNA was transcribed into cDNA with ABScript II RT Mix with genomic DNA Remover (RK20403; Abclonal, Wuhan, China). Fold change of Smurf1 mRNA was detected by SYBR green‐based quantitative real‐time PCR with the primers 5′‐AGATCCGTCTGACAGTGTTATGT‐3′ and 5′‐CCCATCCACGACAATCTTTGC‐3′ purchased from Sangon Biotech, Shanghai, China.

### RNA interference

siRNA targeting p62 (p62 siRNA 2#: F: 5′‐GGCUGAAGGAAGCUGCCUU‐3′ and R: 5′‐AAGGCAGCUUCCUUCAGCC‐3′), targeting PSMC1 (PMSC1 siRNA #2: F: and R: 5′‐GUUCGCUGAAUUUCUCUCUTT‐3′) and control siRNA (Negative Control‐siRNA: F: 5′‐GUACCGCACGUCAUUCGUAUC‐3′ R: 5′‐UACGAAUGACGUGCGGUACGU‐3′) were purchased from JTS scientific. The siRNA was transfected into cells through Lipofectamine RNAiMAX (Invitrogen) following the user guidance. Seventy‐two hours later, the cells were lysed, and the knockdown efficiency of p62 was examined by western blotting.

### Plasmids and molecular cloning agents

Full‐length human Smurf1 and p62 were amplified from the human fetal brain cDNA library. Smurf1 and p62 PCR‐amplified products were subsequently inserted into the p3×Flag‐CMV24 (Sigma) and pEGFP‐N3 (Clontech, Otsu, Shiga Prefecture, Japan) vector at HindIII/SalI sites. The codon‐switched (CS) mutation of pEGFP‐N3‐p62 and site/deletion mutations of p3×Flag‐CMV24‐Smurf1 were generated by Q5 site‐directed mutagenesis kit (New England Biolabs, Beijing, China). All plasmids were confirmed by Sanger sequencing. The restriction enzymes were purchased from New England Biolabs. The DNA polymerase rTaq and Prime STAR high‐fidelity polymerase were purchased from TaKaRa, Kyoto City, Japan. The gel recovery kit was purchased from Thermo Fisher. Plasmid extraction and recovery kit were purchased from TIANGEN. All experiments were operated following the users’ manual.

### Antibodies

The primary antibodies were anti‐(Flag M2) Ig (F3165; Sigma), anti‐β‐actin Ig (A1978; Sigma), anti‐Smurf1 Ig (sc100616; Santa Cruz, Dallas, TX, USA), anti‐(p‐p70 S6 kinase α) Ig (9205, Thr389; CST), anti‐p‐4E‐BP1 Ig (2855s, Thr37/46; CST, Danvers, MA, USA), anti‐p62 Ig (BML‐PW9860; Enzo, Farmingdale, NY, USA), anti‐LC3B Ig (NB100‐2220; Novus, Littleton, CO, USA), anti‐LC3B Ig (L7543; Sigma) and anti‐(nuclear factor erythroid 2‐related factor 2) Ig (Nrf2; 16396‐1‐AP; Proteintech, Chicago, IL, USA).

Secondary antibodies for western blotting were goat anti‐mouse IgG (BA1050; BOSTER) and goat anti‐rabbit IgG (BA1054; BOSTER, Pleasanton，CA，USA). Secondary antibodies for immunofluorescence were Alexa Fluor 488 goat anti‐mouse IgG (A11001; Life Technologies, Carlsbad, CA, USA), Alexa Fluor 555 goat anti‐mouse IgG (A21425; Life Technologies), Alexa Fluor 488 goat anti‐rabbit IgG (A32731; Life Technologies) and Alexa Fluor 647 goat anti‐mouse IgG (ab150115; Abcam, Cambridge, UK). The secondary antibody for immunohistochemical staining was ImmPRESS HRP horse anti‐mouse IgG (MP‐7402; Vector, Stuttgart, Germany).

### Western blotting

Cells were lysed with ice‐cold Radio‐Immunoprecipitation Assay buffer (BOSTER) supplemented with PMSF (BOSTER) and phosphatase inhibitors (BOSTER) and sonicated in ice water with bath sonicator, then boiled in 98 °C dry bath for 10 min. Protein samples (10–100 μg) were then loaded and separated on 4–12% gradient SDS/PAGE and transferred to polyvinylidene difluoride membranes. The membranes were blocked with 5% milk in TBST supplemented with 0.1% Tween 20 for 1 h and then incubated with primary antibodies. Bound antibodies were detected with horseradish peroxidase‐conjugated anti‐rabbit or anti‐mouse secondary antibodies (1 : 5000) and enhanced chemiluminescence reagents.

### Immunofluorescence

After fixation with 4% paraformaldehyde for 20 min and permeabilization in 0.2% Triton X‐100 (PBS) for 5 min, HEK293 cells were incubated with the indicated antibodies for 12 h at 4 °C, followed by incubation with fluorescence‐labeled secondary antibody for 1 h at room temperature in a moist container in the dark. The cells were stained by FluoroShield mounting medium with DAPI, and images were visualized with confocal microscope (Nikon, Tokyo, Japan). The line profile is performed through image j (National Institutes of Health, Bethesda, MD, USA).

### Immunohistochemical staining

GB patient samples were embedded in paraffin and cut into 5‐μm sections, followed by mounting on slides. The slides were treated with xylene and ethanol, then put into 50 mL sodium citrate buffer (41 mL 0.1 m sodium citrate, 9 mL 0.1 m critic acid, add H_2_O to 500 mL), heated in a microwave oven to boiling and then cooled to room temperature. After permeabilization in 0.3% Triton X‐100 (PBS) for 5 min, slides were blocked by 10% goat serum for 1 h at room temperature and incubated overnight at 4 °C with primary antibodies. The ImmPRESS HRP secondary antibodies were applied in slides at room temperature for 1 h. ImmPACT diaminobenzidine (VECTOR) was used for detection, and hematoxylin diaminobenzidine chromogen (Thermo Fisher Scientific, Waltham, MA, USA) was used as a counterstain. Images were taken with Olympus slideview VS200.

### Statistical analysis

Data represent mean ± standard deviation (SD) of *n* = 3 independent experiments. A two‐tailed Student's *t*‐test was used for statistical analysis. The significance level was defined as *P < 0.05, **P < 0.01, ***P < 0.001, or not significant (NS).

## Results

### Smurf1 is degraded by autophagy in GB cells

**Fig. 1:**
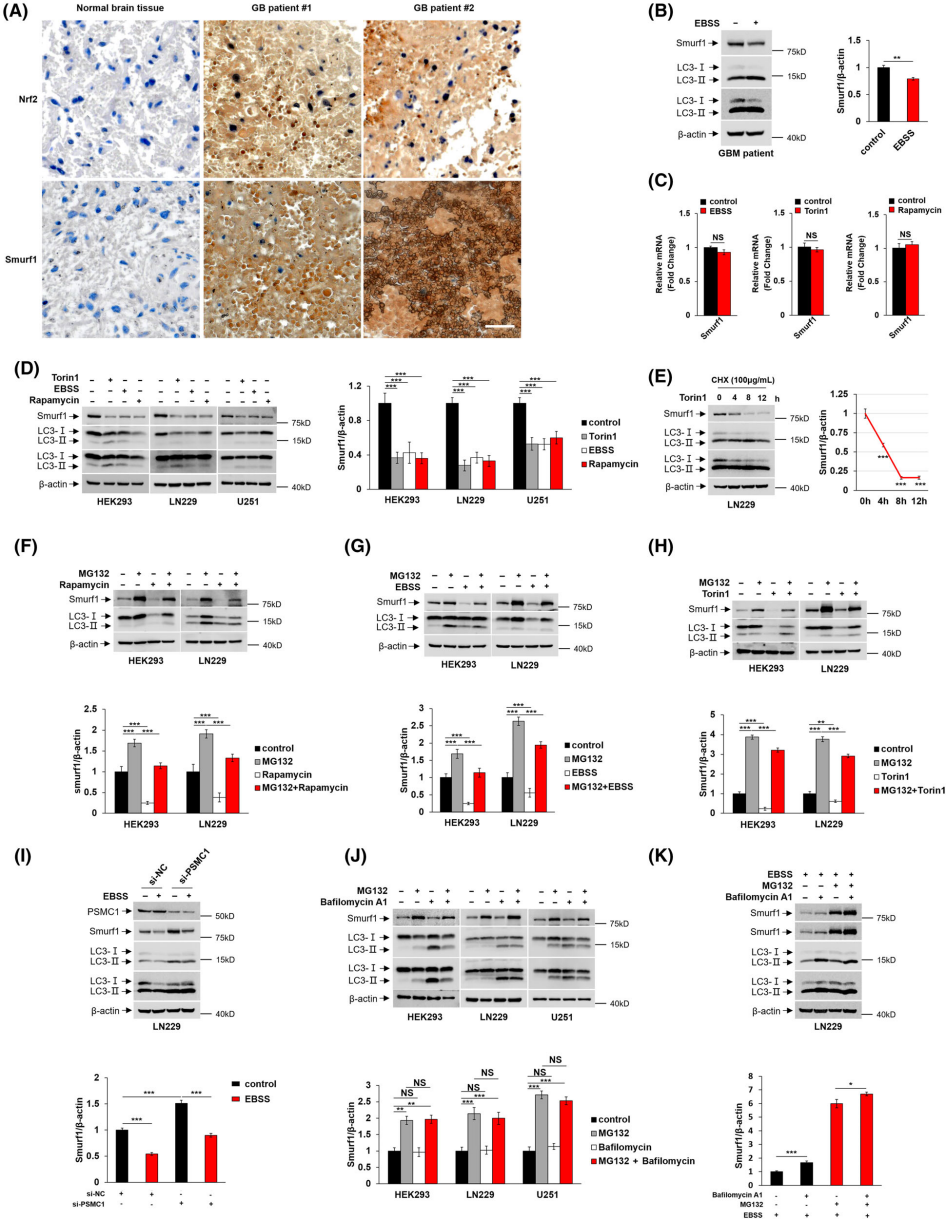
Smurf1 is degraded by autophagy in GB cells. (A) Representative images of immunohistochemical staining of Nrf2 and Smurf1 expression in human normal brain tissues and specimens of patients with GB. Scale bar, 50 μm. (B) Primary cell from patients with GB was starved with Earle''s Balanced Salt Solution (EBSS) and lysed for western blotting with anti‐Smurf1 Ig, anti‐LC3 Ig and anti‐β‐actin Ig. Relative quantification of Smurf1 is shown in the right panel. (C) Total RNA of normal and of autophagy activator‐treated LN229 cells were extracted and reversely transcribed to cDNA for quantitative real‐time PCR to detect the fold change of Smurf1 mRNA. (D) HEK293, LN229 and U251 cell lines were treated with Torin1, EBSS and rapamycin, respectively, and were lysed for western blotting with anti‐Smurf1 Ig, anti‐LC3 Ig and anti‐β‐actin Ig. Relative quantification of Smurf1 is shown in the right panel. (E) LN229 cells were treated with cycloheximide (CHX) and Torin1 in different time periods. Smurf1 degradation rate was quantified in the right panel. (F) HEK293 and LN229 were treated with or without MG132 and/or rapamycin and were lysed for western blotting with anti‐Smurf1 Ig, anti‐LC3 Ig and anti‐β‐actin Ig. Relative quantification of Smurf1 is shown in the lower panel. (G) HEK293 and LN229 were treated with or without MG132 and/or EBSS and were lysed for western blotting with anti‐Smurf1 Ig, anti‐LC3 Ig and anti‐β‐actin Ig. Relative quantification of Smurf1 is shown in the lower panel. (H) HEK293 and LN229 were treated with or without MG132 and Torin1 and were lysed for western blotting with anti‐Smurf1 Ig, anti‐LC3 Ig and anti‐β‐actin Ig. Relative quantification of Smurf1 is shown in the lower panel. (I) Control or PSMC1 siRNA were transfected into LN229 cells with or without EBSS treatment. The cells were lysed for western blotting with anti‐PSMC1 Ig, anti‐Smurf1 Ig, anti‐LC3 Ig and anti‐β‐actin Ig. Relative quantification of Smurf1 is shown in the lower panel. (J) HEK293, LN229 and U251 cell lines were treated with or without MG132 and/or Bafilomycin A1 and were lysed for western blotting with anti‐Smurf1 Ig, anti‐LC3 Ig and anti‐β‐actin Ig. Relative quantification of Smurf1 is shown in the lower panel. (K) LN229 cells were treated with Bafilomycin A1 with or without MG132 under starvation condition. Relative quantification of Smurf1 is shown in the lower panel. The value of the statistical graph is the ratio of the gray value of Smurf1 to β‐actin. Data represent mean ± SD of *n* = 3 independent experiments. A two‐tailed Student's *t*‐test was used for statistical analysis. The significance level was defined as *P* < 0.05: *P < 0.05, **P < 0.01, ***P < 0.001.

The Nrf2 is the best‐characterized antioxidant transcription factor [23]. Thus, the expression level of Nrf2 is regarded as a marker of the oxidative stress of cells. Immunohistochemistry staining shows the expression of Nrf2, and Smurf1 expression is higher in patients with GB compared with normal brain tissue, indicating GB cells are accompanied with overexpression of Smurf1 and stress condition (Fig. 1A). Previously, Smurf1 was known as an oncogene to promote GB progression; thus, it is of significance to study the degradation pathway of overexpressed Smurf1 [9, 24].

To study whether overexpressed Smurf1 is degraded through the autophagy–lysosome pathway, we starved primary cultured cells derived from patients with GB with autophagy activator EBSS. The result showed that the Smurf1 level was decreased after the activation of autophagy (Fig. 1B). To exclude the effect of autophagy activators on transcriptional level, we performed quantitative real‐time PCR to detect the fold changes of Smurf1 mRNA with the treatment of the drugs. We found that the mRNA level of Smurf1 shows no significant difference with or without autophagy activators, which suggests that autophagy activators have no influence on transcriptional levels (Fig. 1C).

We also used different autophagy activators, Torin1, EBSS and rapamycin, to treat cultured HEK293, LN229 and U251. We found the level of Smurf1 is decreased in these three different cell lines checked by western blotting (Fig. 1D), which confirmed that Smurf1 was degraded through autophagy in GB cells. We next treated LN229 with cycloheximide to inhibit protein synthesis and stimulated the cells with Torin1 in increasing time periods. Our data showed that Smurf1 was degraded by autophagy in a time‐dependent manner (Fig. 1E).

In addition, we treated HEK293 and LN229 with autophagy activators under inhibitor MG132 to exclude the effect of the UPS on the degradation of Smurf1. Smurf1 is up‐regulated with the treatment of MG132 in both HEK293 and LN229, indicating blocking the UPS pathway inhibits Smurf1 degradation. The expression of Smurf1 is lower in the group treated with both MG132 and autophagy activators than MG132 treated alone in HEK293 and LN229 cells, further indicating the degradation of Smurf1 is through the autophagy‐mediated pathway (Fig. 1F–H). The 26S protease regulatory subunit 4 (P26s4/PSMC1) is an indispensable ATPase of UPS‐mediated protein degradation [25, 26]. We further performed PSMC1 knockdown to exclude the influence of UPS instead of MG132 treatment. Consistently, we found that Smurf1 expression was higher in the absence of PSMC1 compared with the control siRNA‐transfected group (Fig. 1I). Importantly, we found that Smurf1 has decreased with the starvation of EBSS compared with normal nutritional status in the presence of PSMC1 siRNA, indicating that Smurf1 is degraded by autophagy (Fig. 1I).

We next used MG132 and autophagy inhibitor Bafilomycin A1 to check whether blocking autophagy could increase the Smurf1. Surprisingly, Smurf1 level had no significant difference between Bafilomycin A1 and the control group with or without MG132 treatment in HEK293, LN229 and U251, indicating blocking autophagy did not increase Smurf1 under normal conditions (Fig. 1J). Thus, the observation of autophagic degradation of Smurf1 possibly occurs when autophagy is stimulated. To improve this hypothesis, we treated LN229 cells with Bafilomycin A1 and MG132 under the starvation by EBSS. We found Smurf1 has increased after treatment of Bafilomycin A1 and EBSS compared with the control group (Fig. 1K). Consistently, in the presence of MG132, the Smurf1 level increased with the stimulation of Bafilomycin A1 under the activation of autophagy by EBSS (Fig. 1K). These results suggested that Smurf1 is mainly degraded via the UPS, and autophagic degradation of Smurf1 occurs while autophagy is activated.

### The autophagic degradation of Smurf1 is mediated by p62

**Fig. 2:**
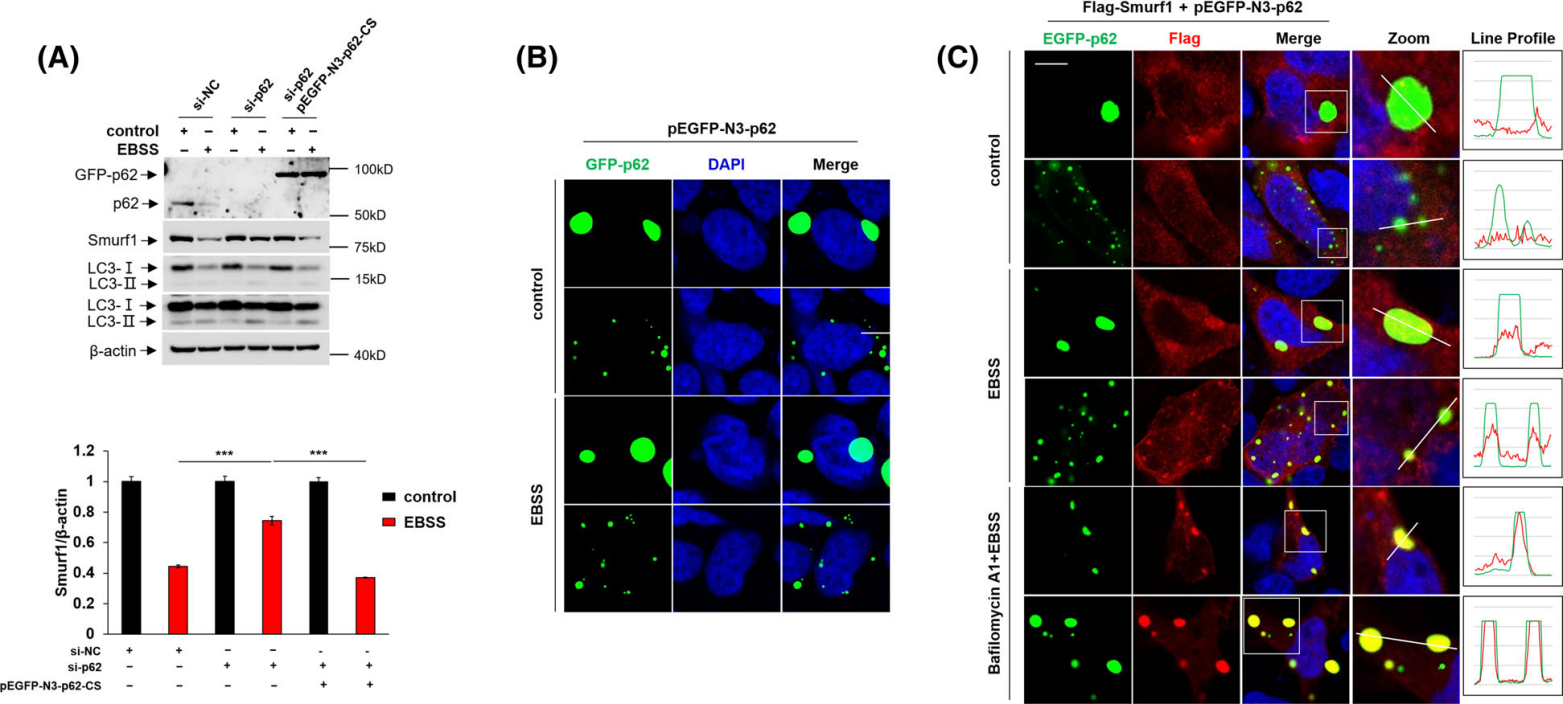
The autophagic degradation of smurf1 is mediated by p62. (A) LN229 cells were transfected with or without si‐NC, si‐p62 and/or pEGFP‐N3‐p62‐CS plasmid and were treated with or without Earle''s Balanced Salt Solution (EBSS), respectively. Cells were lysed for western blotting with anti‐p62 Ig, anti‐Smurf1 Ig, anti‐LC3 Ig and anti‐β‐actin Ig. Relative quantification of Smurf1 is shown in the right panel. The value of the statistical graph is the ratio of the gray value of Smurf1 to β‐actin. Data represent mean ± SD of *n* = 3 independent experiments. A two‐tailed Student's *t*‐test was used for statistical analysis. The significance level was defined as ***P < 0.001. (B) Representative immunofluorescence staining of HEK293 transfected with pEGFP‐N3‐p62 plasmid. Nuclear staining was with 4',6‐diamidino‐2‐phenylindole (DAPI). Green fluorescence is EGFP‐p62 fusion protein. Scale bar, 5 μm. (C) HEK293 cells were transfected with pEGFP‐N3‐p62 and Flag‐Smurf1 (p3×Flag‐myc‐CMV24‐Smurf1) plasmid and treated with or without Bafilomycin A1 under EBSS starvation for representative immunofluorescence staining with anti‐Flag Ig (red). Colocalization of p62 and Smurf1 is illustrated by line profile. Red and green lines indicate Smurf1 and p62, respectively. Scale bar, 5 μm.

Next, we investigated the underlying mechanism of autophagic degradation of Smurf1. The selective autophagy pathway involved several autophagy receptors. One of the well‐known receptors, p62, played an important role in delivering specific cytoplasmic cargo to lysosome for selective degradation [27]. To investigate whether p62 mediates autophagic degradation of Smurf1, we designed and transfected p62 siRNA into LN229 cells with and without EBSS treatment. Consistently, we found that the Smurf1 level is decreased after starvation. Importantly, the autophagic degradation of Smurf1 is inhibited under silencing of p62, which implicates that p62 is required for the autophagic degradation of Smurf1 (Fig. 2A). We also transfected pEGFP‐N3‐p62‐CS (p62‐siRNA‐resistant) plasmid into the si‐p62 LN229 cells with and without EBSS treatment. We found that the Smurf1 level decreased in the p62 overexpression group compared with the p62 knockdown group (Fig. 2A). These results demonstrated that Smurf1 is degraded by autophagy in a p62‐dependent manner.

In addition, we performed immunofluorescence to check colocalization of Smurf1 and p62 on the autophagosome. We transfected pEGFP‐N3‐p62‐CS plasmid into HEK293 cells and found that overexpressed EGFP‐labeled p62 forms puncta with two different sizes in the cytoplasm with or without EBSS treatment (Fig. 2B). The green puncta are autophagosomes formed by p62‐mediated selective autophagy, and bigger puncta may respond to oxidative stress [28]. We further transfected pEGFP‐N3‐p62 and Flag‐Smurf1 plasmids into HEK293 cells and treated them with or without Bafilomycin A1 under EBSS starvation. Significantly, we found that Flag‐labeled Smurf1 forms puncta and colocalizes with EGFP‐labeled p62, demonstrating overexpressed Smurf1 coaggregates with p62 in an aggregate‐like structure (Fig. 2C). The colocalization of Smurf1 and p62 indicates that overexpressed Smurf1 is degraded by p62‐mediated selective autophagy, along with the aggregate‐like cytoplasm cargoes.

### The autophagic degradation of Smurf1 is dependent on its E3 ubiquitin ligase activity

We hypothesized that Smurf1 binds to and ubiquitinates aggregate‐like cargo and is degraded by p62‐mediated selective autophagy. Smurf1 consists of a C2 domain at the N terminus, two WW domains and a homologous to E6AP C‐terminus (HECT) domain at the C terminus. The HECT domain is a catalytic domain associated with Smurf1 ubiquitin ligase activity. The WW domain binds to various proline/tyrosine (PY)‐rich motif (PY motif)‐containing substrates, and the C2 domain is critical for both Smurf1 localization and substrate selection [29, 30, 31]. The highly conserved Cys699 site in the HECT domain is necessary for its catalytic bioactivity. The deletion of the HECT domain or site mutation on Cys699 abolishes the E3 ubiquitin ligase activity of Smurf1 [32, 33]. To further identify whether the Smurf1 autophagic degradation is dependent on its own function, we constructed Flag‐Smurf1‐ΔC2, Flag‐Smurf1‐ΔWW, Flag‐Smurf1‐ΔHECT and Flag‐Smurf1‐C699A plasmids.

**Fig. 3:**
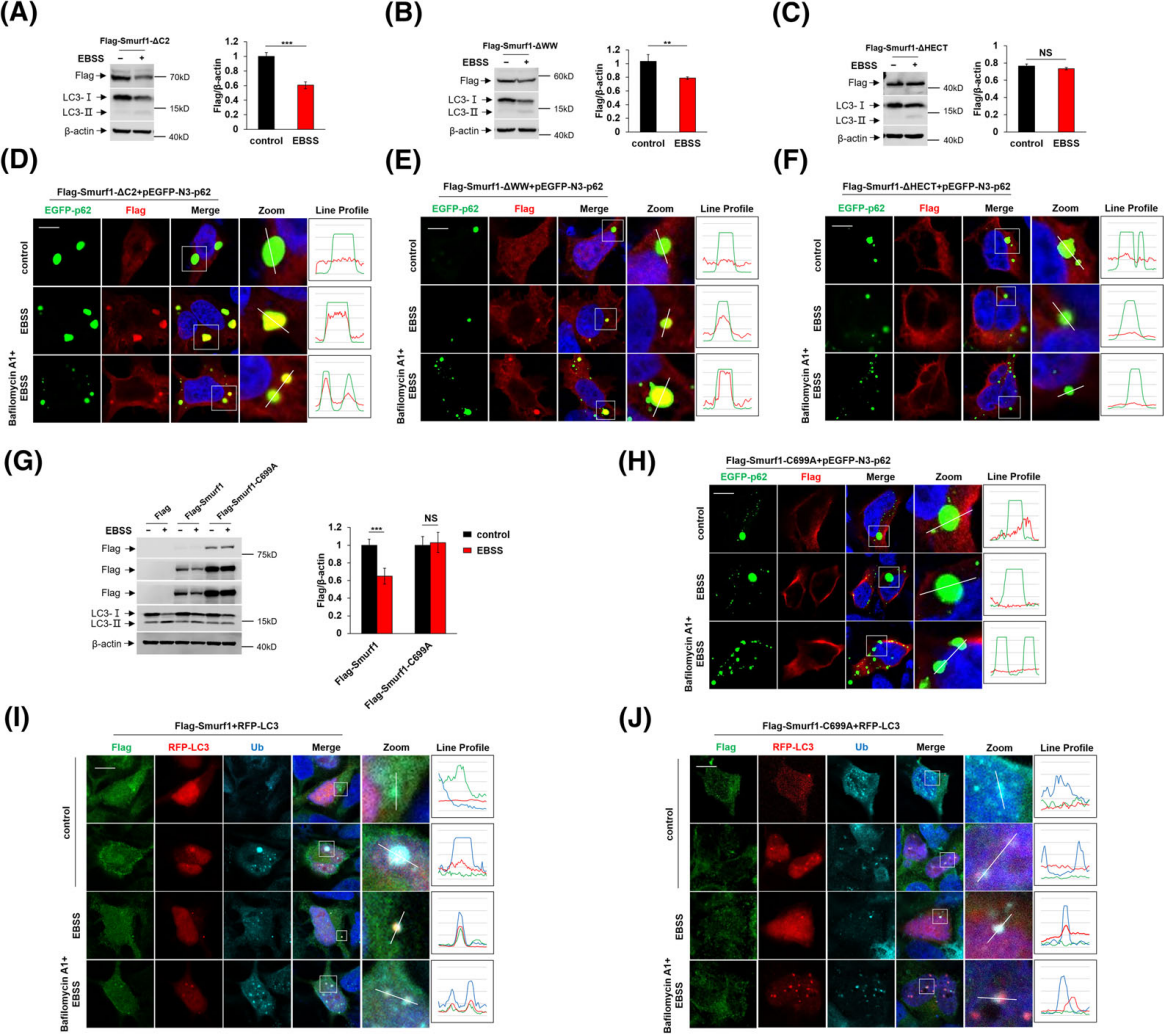
The autophagic degradation of Smurf1 is dependent on its E3 ubiquitin ligase activity. (A) HEK293 cells were transfected with C2 domain deletion construct, Flag‐Smurf1‐ΔC2, and were starved with Earle''s Balanced Salt Solution (EBSS). Cell lysates were examined by western blotting with anti‐Flag Ig, anti‐LC3 Ig and anti‐β‐actin Ig. Relative quantification of Flag‐Smurf1‐ΔC2 fusion protein is shown in the right panel. (B) HEK293 cells were transfected with WW domain deletion construct, Flag‐Smurf1‐ΔWW, and were starved with EBSS. Cell lysates were examined by western blotting with anti‐Flag Ig, anti‐LC3 Ig and anti‐β‐actin Ig. Relative quantification of Flag‐Smurf1‐ΔWW fusion protein is shown in the right panel. (C) HEK293 cells were transfected with homologous to E6AP C‐terminus (HECT) domain deletion construct, Flag‐Smurf1‐ΔHECT, and were starved with EBSS. Cell lysates were examined by western blotting with anti‐Flag Ig, anti‐LC3 Ig and anti‐β‐actin Ig. Relative quantification of Flag‐Smurf1‐ΔHECT fusion protein is shown in the right panel. (D) Representative immunofluorescence staining of HEK293 transfected with pEGFP‐N3‐p62 and Flag‐Smurf1‐ΔC2 plasmids. The cells were treated with or without EBSS and/or Bafilomycin A1 before staining. Colocalization of p62 and Smurf1‐ΔC2 is illustrated by line profile. Red and green lines indicate Smurf1‐ΔC2 and p62, respectively. Scale bar, 5 μm. (E) Representative immunofluorescence staining of HEK293 transfected with pEGFP‐N3‐p62 and Flag‐Smurf1‐ΔWW plasmids. The cells were treated with or without EBSS and/or Bafilomycin A1 before staining. Colocalization of p62 and Smurf1‐ΔWW is illustrated by line profile. Red and green lines indicate Smurf1‐ΔWW and p62, respectively. Scale bar, 5 μm. (F) HEK293 cells are transfected with Flag‐Smurf1‐ΔHECT and pEGFP‐N3‐p62 plasmids. The cells were treated with or without EBSS and/or Bafilomycin A1 before staining. Colocalization of p62 and Smurf1‐ΔHECT is illustrated by line profile. Red and green lines indicate Smurf1‐ΔHECT and p62, respectively. Scale bar, 5 μm. (G) HEK293 cells were transfected with Flag (p3×Flag‐myc‐CMV24), Flag‐Smurf1 (p3×Flag‐myc‐CMV24‐Smurf1) and/or Flag‐Smurf1‐C699A (p3×Flag‐myc‐CMV24‐Smurf1‐C699A), respectively, and were treated with or without EBSS. Cells were lysed for western blotting with anti‐Flag Ig, anti‐LC3 Ig and anti‐β‐actin Ig. Relative quantification of Smurf1 is shown in the right panel. (H) Representative immunofluorescence staining of HEK293 transfected with Flag‐Smurf1‐C699A and pEGFP‐N3‐p62 plasmid. The cells were treated with or without EBSS and/or Bafilomycin A1 before staining. Anti‐Flag Ig was used to stain Flag‐Smurf1 fusion protein. (I, J) Representative immunofluorescence staining of HEK293 transfected with RFP‐LC3 and Flag‐Smurf1 (‐C699A) plasmids. The cells were treated with or without EBSS and/or Bafilomycin A1 before staining. Flag‐Smurf1 (‐C699A) fusion protein was stained by anti‐Flag primary antibody (green) and Alexa Fluor 488 goat anti‐rabbit secondary antibody. Endogenous ubiquitin is stained by anti‐ubiquitin primary antibody (cyan‐blue) and Alexa Fluor 647 goat anti‐mouse secondary antibody. Colocalization of Smurf1 (‐C699A), ubiquitin and LC3 is illustrated by line profile. Red, green and cyan‐blue lines indicate LC3, Smurf1 and ubiquitin, respectively. Scale bars, 5 μm. Data represent mean ± SD of *n* = 3 independent experiments. A two‐tailed Student's *t*‐test was used for statistical analysis. The significance level was defined as *P* < 0.05: **P < 0.01, ***P < 0.001.

To check the role of each domain of Smurf1 in autophagic degradation, we first transfected HEK293 cells with deletion constructs of Smurf1 with or without EBSS starvation. We found that the Flag‐labeled Smurf1‐ΔC2 and Smurf1‐ΔWW are decreased after the activation of autophagy (Fig. 3A,B). However, Smurf1‐ΔHECT could not be down‐regulated after starvation of EBSS, which demonstrates that the HECT domains are necessary for autophagic degradation of Smurf1 (Fig. 3C). We repeated the same experiment in LN229 cells and drew the same conclusion (the data are not shown). In addition, we performed immunofluorescence staining to check the Smurf1 deletion and p62 colocalization by transfecting these three deletion constructs together with pEGFP‐N3‐p62, respectively, into HEK293 cells with or without Bafilomycin A1 under EBSS starvation. Consistently, we found that Flag‐labeled Smurf1‐ΔC2 and Smurf1‐ΔWW, but not Flag‐Smurf1‐ΔHECT, colocalize with EGFP‐labeled p62 puncta under EBSS treatment with no regard to Bafilomycin A1 (Fig. 3D–F). These results indicate that autophagic degradation of Smurf1 is related to its E3 ubiquitin ligase bioactivity.

Next, we transfected the Flag, Flag‐Smurf1 and Flag‐Smurf1‐C699A into the cultured HEK293 cells with or without EBSS treatment. We found that overexpressed wild‐type Smurf1 is decreased after EBSS treatment, but not Smurf1‐C699A, which indicates that autophagic degradation of Smurf1 is inhibited without its E3 ubiquitin ligase activity (Fig. 3G). We also found the same phenomenon in LN229 cells, and the data are not shown. Furthermore, we cotransfected Flag‐Smurf1‐C699A and pEGFP‐N3‐p62 into HEK293 with or without Bafilomycin A1 treatment under EBSS starvation. We found that Smurf1‐C699A cannot form puncta to coaggregate with p62, suggesting that the colocalization of Smurf1 and p62 is blocked without E3 ubiquitin ligase activity (Figs 2C and 3H). These results further demonstrated that autophagic degradation of Smurf1 is dependent on its E3 ubiquitin ligase activity.

LC3 is localized on the membrane of autophagosome [34]. Furthermore, we detected the colocalization of LC3, ubiquitin and Smurf1 with immunofluorescence. We transfected Flag‐Smurf1 plasmid and RFP‐labeled LC3 plasmids into HEK293 cells, treated them by EBSS with or without Bafilomycin A1, and found that Smurf1 forms puncta and colocalizes with both LC3 and ubiquitin. Importantly, we cotransfected the Flag‐Smurf1‐C699A with RFP‐LC3 into HEK293, and we found that Flag‐labeled Smurf1‐C699A cannot colocalize with LC3 and ubiquitin under EBSS treatment (Fig. 3I,J). These data suggest that autophagic degradation of Smurf1 is associated with ubiquitinating aggregate‐like cargo and is inhibited after abolishing the E3 ubiquitin ligase activity. These results demonstrate that Smurf1 is degraded by autophagy together with the ubiquitinated cytoplasmic cargo in the ubiquitin ligase activity‐dependent manner.

### Autophagy degrades Smurf1 to inhibit the PI3K/Akt signaling pathway in GB cells

The PI3K/Akt signaling pathway is one of the most frequently dysregulated pathways in cancer and promotes cell proliferation [35, 36]. It is known to promote cell proliferation through phosphorylation of p70S6k and 4E‐BP1. The 70‐kDa ribosomal S6 kinase (p70S6k) is a downstream effector of mammalian target of rapamycin complex 1 and a regulator of cell proliferation [37]. Translational repressor 4E‐BP1 is another downstream effector of PI3K/Akt signaling, which blocks the translation of growth‐promoting genes [38]. In this study, we hypothesized that autophagic degradation of overexpressed Smurf1 down‐regulates phosphorylation levels of Akt, p70S6k and 4E‐BP1. The promoting role of Smurf1 in regulating the PI3K/Akt signaling pathway has been clarified [9]; however, whether autophagy affects the transduction of the PI3K/Akt signaling pathway by degrading Smurf1 remains unknown.

**Fig. 4:**
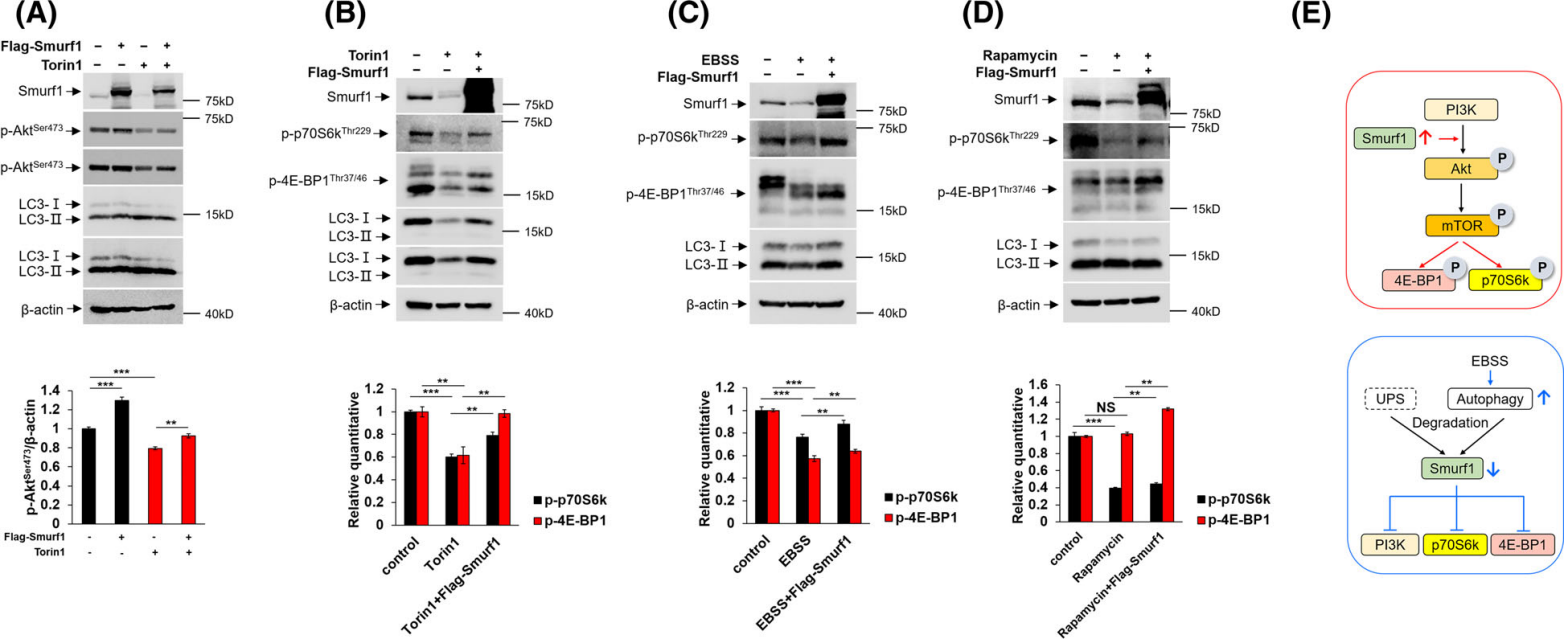
Autophagy degrades Smurf1 to inhibit the PI3K/Akt signaling pathway in GB cells. (A) LN229 cells were transfected with Flag‐Smurf1 plasmid and treated with or without Torin1. The cells were lysed for western blotting with anti‐p‐Akt^Ser473^ Ig, anti‐Smurf1 Ig, anti‐LC3 Ig and anti‐β‐actin Ig. The relative quantity of p‐Akt^Ser473^ is shown in the lower panel. (B) LN229 cells were treated by Earle''s Balanced Salt Solution (EBSS) and lysed for western blotting. Another group of cells was first transfected with Flag‐Smurf1 plasmids and was treated with EBSS. All the cell lysates were examined by western blotting with anti‐Smurf1 Ig, anti‐LC3 Ig, anti‐β‐actin Ig, anti‐p‐p70S6k^Thr229^ Ig and anti‐p‐4E‐BP1^Thr37/46^ Ig. The relative quantifications of p‐p70S6k^Thr229^ and p‐4E‐BP1^Thr37/46^ are shown in the lower panel. (C) LN229 cells were treated with or without Torin1 for 36 h. After 12 h, Flag‐Smurf1 plasmid was transfected into the third group of cells. Torin1 was always supplemented in culture medium until the cells were lysed for western blotting. Anti‐Smurf1 Ig, anti‐LC3 Ig, anti‐β‐actin Ig, anti‐p‐p70S6k^Thr229^ Ig and anti‐p‐4E‐BP1^Thr37/46^ Ig were used. The relative quantifications of p‐p70S6k^Thr229^ and p‐4E‐BP1^Thr37/46^ are shown in the lower panel. (D) LN229 cells were treated with or without rapamycin for 36 h. After 12 h, Flag‐Smurf1 plasmid was transfected into the third group of cells. Rapamycin was always supplemented in culture medium until the cells were lysed for western blotting. Anti‐Smurf1 Ig, anti‐LC3 Ig, anti‐β‐actin Ig, anti‐p‐p70S6k^Thr229^ Ig and anti‐p‐4E‐BP1^Thr37/46^ Ig were used. The relative quantifications of p‐p70S6k^Thr229^ and p‐4E‐BP1^Thr37/46^ are shown in the lower panel. (E) Model of autophagy degrades Smurf1 in a p62‐dependent manner and inhibits transduction of PI3K/Akt signaling in GB cells. The value of the statistical graph is the ratio of the gray value of phosphor‐p70S6k^Thr229^ (or phosphor‐4E‐BP1^Thr37/46^) to β‐actin. Data represent mean ± SD of *n* = 3 independent experiments. A two‐tailed Student's *t*‐test was used for statistical analysis. The significance level was defined as *P* < 0.05: **P < 0.01, ***P < 0.001.

In our study, we found that overexpression of Smurf1 increases the phosphorylation level of Akt, whereas treating cells with Torin1 alone decreases the protein level of phosphor‐Akt^Ser473^ (Fig. 4A). Importantly, overexpressing Smurf1 under Torin1 treatment increases Akt phosphorylation compared with the Torin1 alone treatment group, which suggests that Smurf1 promotes the transduction of the PI3K/Akt signaling pathway, and autophagy inhibits this pathway by degrading Smurf1 (Fig. 4A). We further treated the LN229 cells with autophagy activators with or without Smurf1 overexpression and checked the expression of phosphor‐p70S6k^Thr229^ and phosphor‐4E‐BP1^Thr37/46^. We found that phosphor‐p70S6k^Thr229^ and phosphor‐4E‐BP1^Thr37/46^ have decreased with the treatment of EBSS or Torin1 in LN229 (Fig. 4B–D). We next transfected Flag‐Smurf1 plasmid into LN229, treated the cells with EBSS or Torin1, and found that the protein levels of phosphor‐p70S6k ^Thr229^ and phosphor‐4E‐BP1^Thr37/46^ increased (Fig. 4B–D). It further confirms that Smurf1 plays an important role in the PI3K/Akt signaling pathway, and autophagic degradation of Smurf1 inhibits the transduction of the PI3K/Akt signaling pathway in GB cells. Previously, the level of phosphor‐4E‐BP1^Thr37/46^ did not change with the treatment of rapamycin, because Thr37 and Thr46 sites of 4E‐BP1 are not sensitive to rapamycin in glioma cells [39, 40]. We also used mammalian target of rapamycin inhibitor rapamycin to treat the LN229 cells. We found that phosphor‐p70S6k^Thr229^, but not phosphor‐4E‐BP1^Thr37/46^, has decreased with the treatment of rapamycin (Fig. 4D). Next, we transfected Flag‐Smurf1 plasmid into the cells with rapamycin treatment and found that phosphor‐p70S6k ^Thr229^ and phosphor‐4E‐BP1^Thr37/46^ levels increase (Fig. 4D). Notably, overexpression of Flag‐Smurf1 did not always restore the phosphorylation of Akt, p70S6K and 4E‐BP1 back to the level similar to the control lane under the endogenous condition, and the possible reason is that the stimulation of drugs and transfection reagents has a negative impact on the growth of cells. Collectively, these results further confirm that autophagy degrades Smurf1 to inhibit the transduction of the PI3K/Akt signaling pathway. The inhibition of the PI3K/Akt pathway through autophagy may block tumor proliferation and survival.

## Discussion

How to degrade overexpressed Smurf1 in tumor cells and to maintain homeostasis remain largely unknown. The degradation of Smurf1 through UPS has been well established; in this study, we found that autophagosomal machinery plays a vital role in degrading excessive Smurf1. We discussed the underlying mechanism of autophagic degradation, which is E3 ubiquitin ligase function and p62 dependent. Autophagy is a well‐known mechanism that degrades large aggregates; thus, we hypothesized that the degradation of Smurf1 molecule is mainly carried out by the UPS, and Smurf1 possibly binds to and ubiquitinates cytoplasm cargoes for p62‐mediated selective autophagic degradation together with the aggregates. We further confirmed that autophagy degrades Smurf1 to inhibit transduction of the PI3K/Akt signaling pathway (Fig. 4E).

First, excess Smurf1 is degraded through p62‐mediated autophagy. Misfolded proteins form aggregates and are delivered to autophagosomes for degradation by selective autophagy receptors. According to previous studies, autophagy receptor p62 clustered with ubiquitinated proteins and delivered the aggregates to autophagosome [22, 41, 42]. The interaction between p62 and E3 ubiquitin ligase has been reported [20, 43]. We used p62‐siRNA to check whether the autophagic degradation of Smurf1 is p62 dependent, and we found that autophagic degradation of Smurf1 is inhibited without p62. We also performed immunofluorescence and found that Smurf1 colocalizes with p62 on the autophagosome. These results suggest that Smurf1 is degraded by p62‐mediated selective autophagy.

Second, the autophagic degradation of Smurf1 is dependent on its E3 ubiquitin ligase activity. We investigated whether the E3 ubiquitin ligase activity of Smurf1 is involved in its autophagic degradation, because Smurf1 is possibly degraded by p62‐mediated selective autophagy together with the ubiquitinated protein aggregates [42]. We hypothesized that Smurf1 is associated with the ubiquitination of protein aggregates. We used deletion constructs of Smurf1 for western blotting and immunofluorescence, and the results suggested the catalytic domain (HECT domain) is necessary for autophagic degradation of Smurf1. We found that mutation of highly conserved cysteine in the HECT domain leads to inhibition of Smurf1 autophagic degradation, and this site mutation dysfunctions E3 ubiquitin ligase activity of Smurf1. Next, we performed immunofluorescence and found that Smurf1 colocalized with LC3 and ubiquitin, which directly indicated that ubiquitination was involved in the autophagic degradation of Smurf1. However, the abolishment of E3 ubiquitin ligase activity of Smurf1 disabled the colocalization among Smurf1, LC3 and ubiquitin, suggesting that the E3 ubiquitin ligase activity of Smurf1 was necessary for its autophagic degradation. In summary, Smurf1 possibly binds to the aggregate‐like cytoplasm cargo and catalyzes ubiquitination of this autophagy substrate; then p62 interacts with the ubiquitinated cargo to deliver the substrate for selective autophagic degradation (Fig. 4E).

Autophagy degrades Smurf1 to inhibit the PI3K/Akt signaling pathway in GB cells. We found that PI3K/Akt signaling was inhibited with the treatment of autophagy activators in GB cells. After overexpressing Smurf1 plasmids in the GB cells treated with autophagy activators, we found that PI3K/Akt signaling pathways were reactivated. Thus, we concluded that overexpressed Smurf1 in GB cells promotes hyperactivation of PI3K/Akt signaling pathways and autophagy degrades Smurf1 to inhibit the transduction of the PI3K/Akt signaling pathway. In summary, our study shows that autophagy degrades overexpressed Smurf1 in GB cells, and the enhancement of autophagy in high Smurf1 expression tumor cells could be a potential antitumor approach. Further studies are required to validate whether inhibition of Smurf1 through the activation of autophagy is effective for tumor treatment.

## Conflict of interest

The authors declare no conflict of interest.

### Author contributions

Lei Dong and Qin Xia designed the experiments and guided the work. Da Han and Shengzhen Li performed the experiments and acquired the data. Da Han analyzed the data and drafted the paper. Lei Dong and Xinyi Meng revised the paper.

## Data Availability

The data that support the findings of this study are available in the figures of this article.
